# A Natural Bacterial-Derived Product, the Metalloprotease Arazyme, Inhibits Metastatic Murine Melanoma by Inducing MMP-8 Cross-Reactive Antibodies

**DOI:** 10.1371/journal.pone.0096141

**Published:** 2014-04-30

**Authors:** Felipe V. Pereira, Carla A. Ferreira-Guimarães, Thaysa Paschoalin, Jorge A. B. Scutti, Filipe M. Melo, Luis S. Silva, Amanda C. L. Melo, Priscila Silva, Manoela Tiago, Alisson L. Matsuo, Luiz Juliano, Maria A. Juliano, Adriana K. Carmona, Luiz R. Travassos, Elaine G. Rodrigues

**Affiliations:** 1 Department of Microbiology, Immunology, and Parasitology, Escola Paulista de Medicina (EPM), Universidade Federal de São Paulo (UNIFESP), São Paulo, Brazil; 2 Department of Biophysics, EPM-UNIFESP, São Paulo, Brazil; 3 School of Pharmaceutical Sciences, University of São Paulo (USP), São Paulo, Brazil; University of Pécs Medical School, Hungary

## Abstract

The increased incidence, high rates of mortality and few effective means of treatment of malignant melanoma, stimulate the search for new anti-tumor agents and therapeutic targets to control this deadly metastatic disease. In the present work the antitumor effect of arazyme, a natural bacterial-derived metalloprotease secreted by *Serratia proteomaculans*, was investigated. Arazyme significantly reduced the number of pulmonary metastatic nodules after intravenous inoculation of B16F10 melanoma cells in syngeneic mice. *In vitro*, the enzyme showed a dose-dependent cytostatic effect in human and murine tumor cells, and this effect was associated to the proteolytic activity of arazyme, reducing the CD44 expression at the cell surface, and also reducing *in vitro* adhesion and *in vitro/in vivo* invasion of these cells. Arazyme treatment or immunization induced the production of protease-specific IgG that cross-reacted with melanoma MMP-8. *In vitro*, this antibody was cytotoxic to tumor cells, an effect increased by complement. *In vivo*, arazyme-specific IgG inhibited melanoma lung metastasis. We suggest that the antitumor activity of arazyme in a preclinical model may be due to a direct cytostatic activity of the protease in combination with the elicited anti-protease antibody, which cross-reacts with MMP-8 produced by tumor cells. Our results show that the bacterial metalloprotease arazyme is a promising novel antitumor chemotherapeutic agent.

## Introduction

Melanoma is a fatal skin cancer, with increased incidence in recent years [1], [2]. Despite improvements in awareness and early detection, the mortality in patients with melanoma is still quite high [3]. A median survival of 8–18 months after diagnosis of metastatic melanoma has been observed [4]. Until 2011, only dacarbazine and high-dosage of interleukin-2 had been approved for melanoma treatment by the Food and Drug Administration (FDA), with durable responses in some patients with metastatic disease [5], [6]. Recently, the newly approved therapies, ipilimumab (anti-CTLA-4 antibody) and vemurafenib (B-RAFV600E kinase inhibitor), have shown a survival benefit in large randomized clinical trials, but still with low frequency of objective results [7], [8]. Chemotherapy used against metastatic melanoma often generates a large number of adverse side effects, leading to interruption of the treatment [9].

The discovery and introduction of new therapeutic agents and strategies is thus actively encouraged in order to expand the few treatment options for metastatic melanoma, and there has been a long-standing interest in the identification of plant- and bacterial-derived natural products for developing anticancer agents.

Exogenous proteinases administered in the form of a multienzyme mixture composed of trypsin, chymotrypsin and papain, effectively inhibited tumor growth in experimental models [10]. Mice treated with bromelain (an extract containing a mixture of proteolytic enzymes prepared from pineapples, *Ananas comosus*) and fastuosain (a 25 kDa cysteine protease purified from the unripe fruits of *Bromelia fastuosa*) were equally protective against tumor development [11]. The antitumor activities of bacterial-derived proteases are less recognized.

Inhibition of endogenous matrix metalloproteases (MMPs) *in vivo* could be a target for cancer treatment. MMPs are linked to invasion and metastasis of tumor cells mediating extracellular matrix (ECM) disruption, and recently they have also been implicated in tumor growth and angiogenesis [12]. However, metalloprotease inhibitors (e.g. metal chelators) are not specific and could affect normal enzymatic reactions. Recent evidence has shown that inhibited secretion of MMPs reduced tumor cell migration and angiogenesis [13], [14]. Moreover, blockade of MMP-14 by a monoclonal antibody in MMP-14-expressing ovarian tumor cells, inhibited aggressive metastatic tumor development in a preclinical model [15].

Arazyme is a 51.5 kDa metalloprotease secreted by *Serratia proteamaculans*, a symbiotic bacterium from *Nephila clavata* spider. Large amounts of the enzyme can be obtained per liter of bacterial culture (in order of grams), the enzymatic activity being maintained under aggressive conditions [16], [17]. A hepatoprotective effect of arazyme was shown in the model of acute liver injury induced by CCl_4_, leading to overexpression of SMP30, inhibition of TGF-β/Smad pathway and increased expression of antioxidant proteins [18].

In the present work we show that arazyme has a potent inhibitory effect on metastatic melanoma B16F10 preclinical model *in vivo*. This effect was attributed to a direct action of arazyme on tumor cells, in association with the induction of protease-specific antibodies recognizing the melanoma MMP-8, that may target this enzyme in the tumor cell environment, both actions interfering with melanoma development.

## Materials and Methods

### Cell lines and culture conditions

The murine melanoma cell line B16F10-Nex2, syngeneic to C57Bl/6 mice, was established at the Experimental Oncology Unit, Paulista School of Medicine, Federal University of São Paulo (EPM-UNIFESP), as described elsewhere [19]. Human melanoma cell line A2058 (CRL-11147, ATCC) and human breast carcinoma SKBR3 (HTB-30, ATCC) were donated by the Ludwig Institute for Cancer Research, São Paulo, Brazil. Human cervical carcinoma (HeLa, CCL-2, ATCC) cell line was gifted by Dr. Hugo P. Monteiro, EPM-UNIFESP. Cells were maintained in culture flasks at 37°C in a humidified atmosphere with 5% CO_2_ in RPMI 1640 medium (pH 7.2; Invitrogen, USA) supplemented with 10 mM HEPES (N-2-hydroxyethylpiperazine-N-2-ethanesulphonic acid), 24 mM sodium bicarbonate, 10% fetal calf serum (FCS, all from Invitrogen, NY, USA) and 40 µg/mL gentamicin (Hipolabor Farmaceutica, MG, Brazil).

### Animals

Inbred male C57Bl/6 mice, 6–8 weeks old, and male albino rabbits, 6 weeks old, were purchased from Center for Development of Experimental Models (CEDEME), at UNIFESP. All animal experiments were approved by the Animal Experimentation Ethics Committee, UNIFESP, under the protocol number 0288/12.

### Arazyme purification and determination of proteolytic activity

The supernatant of *S. proteamaculans* culture medium, obtained from Insect Biotech, Korea, was subjected to membrane filtration and concentrated 3–10 times through 10 kDa cut-off membranes. Protease purification was performed by ion exchange chromatography in a Resource Q column (1 mL, GE Healthcare, Piscataway, NJ, USA) equilibrated with 20 mM Tris-HCl, pH 8.0 and eluted with a gradient of NaCl (0 to 0.5 M), using a Akta Purifier system (GE Healthcare, Uppsala, Sweden). The profile of protein elution was monitored by UV absorbance (280 nm). Fractions of 1 mL were collected at a flow rate of 1 mL/min and protease activity was measured using the synthetic fluorescence resonance energy transfer (FRET) peptide Abz-KLRFSKQ-EDDnp, as described in [16]. Briefly, the test was performed in 50 mM Tris-HCl, pH 8.0 at 37°C, and fluorescence was continuously monitored at λ_ex_ = 320 nm and λ_em_ = 420 nm (1.0 mL final volume) in a Hitachi F-2000 spectrofluorometer (Tokyo, Japan). The inactivated enzyme was obtained by incubation of the purified arazyme at 50°C for 30 min, or by incubation with 2 mM of *ortho-*phenantroline for 5 min. Both treatments inhibited 100% of protease activity, as described previously [16].

### Cell viability assay

A2058, HeLa and B16F10-Nex2 tumor cells were plated into 96-well plates (10^3^ cells/100 µL of medium/well) and treated with increasing concentrations of active or inactive arazyme (in 100 µL of medium) for 24 or 48 hours. Total cells, in the supernatant and adherent cells collected after 0.05% EDTA treatment, were counted in presence of Trypan blue. The percentage of viable and nonviable cells was calculated compared to untreated cells, considered as 100%.

### Adhesion assay

For the adhesion assay, A2058 and B16F10-Nex2 cells (5×10^4^ cells/well) were treated with arazyme (10 µg/mL) for 1 hour, added to 96-well plates and incubated for 3 hours at 37°C. Plates were gently washed twice with PBS to remove unattached cells and the attached cells were fixed with methanol on ice for 5 min. Fixed cells were stained with toluidine blue 1% in sodium tetraborate 1% for 5 min and washed with PBS. Dye was solubilized in SDS 1% for 20 min at 37°C and the resulting colored solution was quantified at 540 nm using a scanning multiwell spectrophotometer. Cells incubated without arazyme were used as control and represent one hundred percent of adhesion.

### Matrigel Invasion Assay

Arazyme effect on B16F10-Nex2 cell invasion was determined as described elsewhere [11] http://www.ncbi.nlm.nih.gov/pubmed/17898868. Briefly, 56 µL (50 mg) of 1∶3 (vol∶vol) serum-free RPMI-diluted cold Matrigel (Basement Membrane Matrix, BD Biosciences, NJ, USA) was added to the upper transwell chambers (8-mm pore size, Corning Costar Co., MA, USA) and incubated for 30 min at 37°C for gel formation. The lower chambers were filled with FCS-containing RPMI medium. Tumor cells (2×10^5^/mL) were treated with arazyme (5 or 10 µg/mL) in serum-free RPMI medium for 1 hour at 37°C and 5% CO_2_, washed, resuspended in 0.2 mL of serum-free RPMI, added to the upper transwell compartment and incubated for 5 hours at 37°C, 5% CO_2_. After removal of non-invading cells with a cotton swab from the top of the membrane, cells underneath the membrane filter were fixed in paraformaldehyde (3.7%) for 15 min, stained with 0.1% toluidine blue solution for 2 min at 37°C and after washing with tap water, the filters were incubated with 200 µL of 1% SDS solution for 1 hour at 37°C. This solution was transferred to a 96-well ELISA plate, and absorbance was measured at 600 nm. The percentage of invasion was calculated compared to the untreated control, taken as 100%.

### Flow Cytometry analysis of CD44 on tumor cells

B16F10-Nex2 or A2058 tumor cells (10^6^ cells/well in 24-well plates) were incubated with arazyme at different concentrations, treated or not with *ortho*-phenantroline for inactivation, in serum-free RPMI medium for 1 hour at 37°C. Cells were collected, transferred to a 1.5-mL microtube and after enzyme removal by PBS washing cells were resuspended in PBS containing 10% BSA and incubated for 10 min on ice. After washing, 1 µg of FITC-conjugated antibody against mouse or human CD44 (BD Biosciences, San Jose, CA) was diluted in 50 µL of PBS containing 1% BSA and added to the cells. After incubation on ice for 1 hour protected from light cells were washed and resuspended in 2% cold paraformaldehyde (wt/vol). Fluorescence was measured on a FACScan flow cytometer (BD Biosciences) and data were analyzed by CellQuest software (Becton Dickinson, San Jose, CA).

### CD44 mRNA quantification by real-time PCR

CD44 mRNA expression from 10^6^ B16F10-Nex2 and A2058 cells treated or not with arazyme (10 µg/mL) for 1 hour was analysed by real-time PCR. Total RNA extraction was performed using TRIzol reagent (Gibco-BRL, NY, USA) composed of a monophasic solution of phenol and guanidine isothiocyanate, according to the method described by Chomczynski and Sacchi [20]. Extracted RNA was quantified using a Nanodrop 2000 Spectrophotometer (Thermo Scientific, MA, USA) and read at 260 nm and 280 nm. All samples with A260/280 greater than 1.8 were considered adequate for the experiments. The synthesis of cDNA was performed using High Capacity cDNA Reverse Transcription Kit (Applied Biosystems, NY, USA) following manufacturer's instructions. Specific mRNA expression was assessed by SYBR Green real-time PCR using 100 ng of cDNA total, Universal SYBR Green Master Mix (Applied Biosystems), and the following pairs of primers in separate reactions: murine CD44 (forward 5′ CATCGAGAAGAGCACCCCAG 3′, reverse 5′ TGAGTGCACAGTTGAGGCAA 3′), human CD44 (forward 5′ TCCCAGACGAAGACAGTCCCTGGAT 3′, reverse 5′ CACTGGGGTGGAATGTGTCTTGGTC 3′), human GAPDH (forward 5′ TGCACCACCAACTGCTTAGC 3′, reverse 5′ GGCATGGACTGTGGTCATGAG 3′) and murine HPRT (forward 5′GCTGGTGAAAAGGACCTCT 3′, reverse 5′CACAGGACTAGAACACCTGC 3′). CD44, GAPDH and HPRT mRNA expressions were obtained from the cycle threshold (Ct) associated with the exponential growth of the PCR products. Quantitative values for CD44 mRNA expression were obtained by the parameter 2^–ΔΔCt^, in which ΔCt represents the subtraction of the GAPDH or the HPRT Ct values from the CD44 Ct values.

### Production, purification and detection by ELISA of polyclonal monospecific arazyme-specific antibodies

C57Bl/6 mice were treated i.p. with arazyme (3 mg/kg/dose) every other day for 21 days. Serum was collected 3 days after the last injection and arazyme binding specificity of serum antibodies was evaluated by ELISA. Briefly, high-binding ELISA plates (Nunc, Thermo Fisher Scientific, NY, USA) were coated with 1 µg of arazyme. After blocking, plates were incubated with serial dilutions of individual sera, 1∶100 to 1∶800. Reaction was revealed with Horseradish Peroxidase (HRP)-conjugated anti-mouse IgG secondary antibodies and DAB (3,3′-Diaminobenzidine tetrahydrochloride), and read in a Multiskan ELISA reader at 492 nm. Additionally, mouse IgG fraction was affinity-purified from pooled sera using a Protein G column (Hi-Trap Protein G affinity column, Amersham Biosciences, Piscataway, NJ).

Male albino rabbits were immunized subcutaneously with 6 doses of 100 µg of arazyme emulsified in alum as adjuvant (v/v, Sigma-Aldrich, MO, USA) every 15 days. Before each immunization serum samples were collected to evaluate the production of arazyme-specific immunoglobulins by ELISA. The serum was inactivated by incubation at 56°C for 30 min, and stored at −80°C in aliquots of 500 µL until purification of antibodies by Protein G affinity chromatography.

### Western blot

B16F10-Nex2 cell lysate (3×10^7^ cells) was prepared by several rounds of freezing in liquid nitrogen and rapid thawing at 37°C. For immunoblot analysis, 40 µg of total tumor cell protein, 100 µg of recombinant murine matrix metalloprotease 1, 2, 7, 8, 9, 11 and 20 (293T Lysate, Santa Cruz Biotechnology, CA, USA) or 10 µg of arazyme were separated in 10% SDS-PAGE and transferred to a nitrocellulose membrane (Millipore, Billerica, MA). The membranes were washed in Tris-buffered saline with Tween 20 (TBS-T, 10 mM Tris-HCl, pH 8, 150 mM NaCl and 0.05% Tween 20) and blocked with 5% skimmed milk (Molico, Nestle, São Paulo, Brazil) in TBS-T for 16 hours at 4°C with shaking. Membranes were then probed for 16 hours at 4°C with primary antibodies specific for detection of arazyme (rabbit polyclonal arazyme antibody produced as described above, diluted 1∶200), or anti-murine MMP-1, MMP-2, MMP-7, MMP-8, MMP-9, MMP-11 or MMP-20 (Santa Cruz Biotechnology). After 1 hour incubation with 1∶1,000 rabbit peroxidase-conjugated secondary antibody (Invitrogen), the immunoreactive proteins were detected by enhanced chemiluminescence using ECL detection system (GE Healthcare).

### Immunoprecipitation

Total cell lysate of B16F10-Nex2 cells prepared by freeze/thawing method as described above (500 µg of protein) was incubated for 16 hours with 20 µg of rabbit antibody anti-arazyme at 4°C with gentle shaking. Protein G-Sepharose (500 µL, Amersham Biosciences) was added to the sample and incubated at the same conditions. Beads were collected by centrifugation at 3,000 rpm for 5 min at 4°C and washed twice with PBS-0.05% Tween 20 and once with PBS. Immunoprecipitated proteins were dissolved by boiling in SDS gel loading buffer, separated from the beads by centrifugation and subjected to Western blot as described above.

### *In vitro* cytotoxic effect mediated by arazyme-specific purified IgG

B16F10-Nex2 cells (5×10^3^/100 µL) were cultivated in 96-wells plate for 12 hours. Mouse arazyme-specific purified IgG or irrelevant mouse IgG [21] were added at different concentrations, with or without guinea-pig complement (1∶80, Invitrogen). Viable cells were counted after 24 hours in a hemocytometer in presence of Trypan blue, and the frequency calculated compared to the untreated control.

### *In vivo* assays

C57Bl/6 mice were i.v. injected with 5×10^5^ B16F10-Nex2 melanoma cells, in the caudal vein. Starting on the 1^st^ day after tumor cell inoculation, active arazyme (3 mg/kg) or PBS was administered i.p. every other day for 21 days. Pulmonary metastatic nodules were counted using an inverted microscope on the 22^nd^ day.

For neutralization assay, B16F10-Nex2 melanoma cells (3×10^6^ cells/mL) were incubated in 1.5-mL microtubes for 1 hour at 37°C, 5% CO_2_ and gentle shaking, with PBS (control), 20 µg/mL of active or *ortho-*phenantrolyne-inactivated arazyme (2 mM, 5 min). After three PBS washings, C57Bl/6 mice were injected i.v. with 3×10^5^ tumor cells in serum-free RPMI, and pulmonary metastatic nodules were counted 13 days after tumor cell inoculation.

For treatment with arazyme-specific polyclonal antibodies, mice were inoculated i.v. with 3×10^5^ B16F10-Nex2 cells and 24 hours later, animals were treated i.p. with 0.3 mL antiserum from arazyme-immunized rabbit, 0.3 mL of rabbit pre-immune serum, or PBS. After 13 days the number of metastatic melanotic nodules was counted.

### Statistical analysis

The data are represented as means ± SE. Statistical analysis was performed using Student's t Test. Values (p) equal to or less than 0.05 were considered significant. All experiments were conducted two or more times. Reproducible results were obtained and representative data are shown.

## Results

### Arazyme treatment significantly reduces lung melanoma metastasis

**Figure 1:**
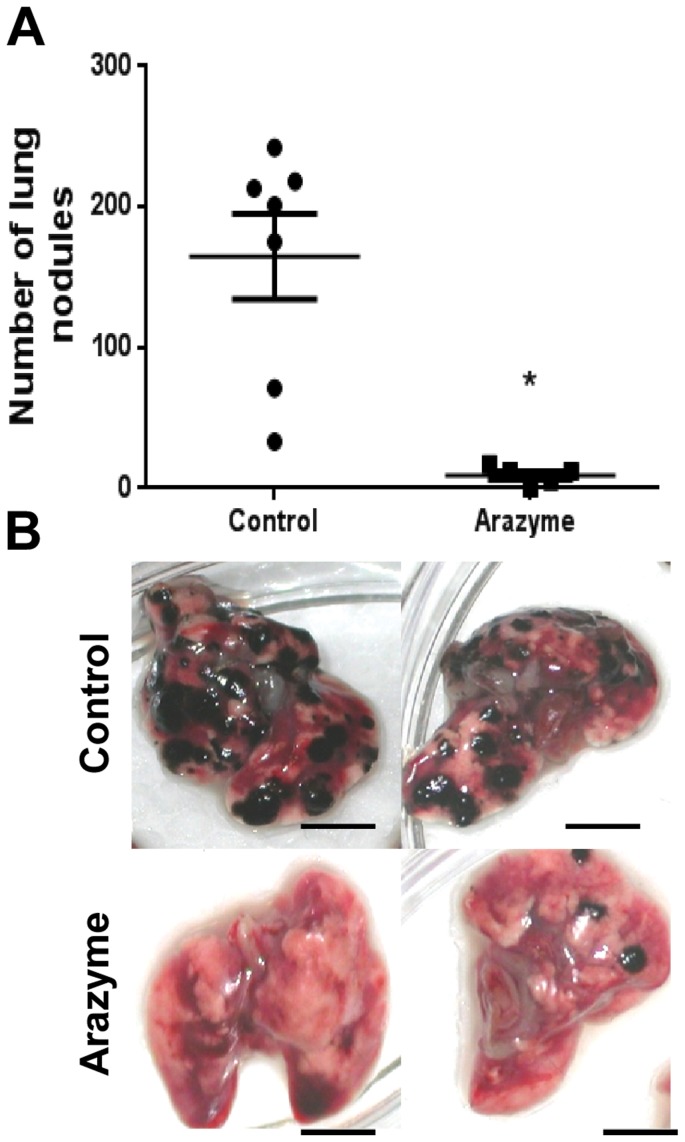
Active arazyme reduces the number of pulmonary metastatic nodules in the murine melanoma model. (**A**) C57Bl/6 mice were i.v. injected with 5×10^5^ B16F10-Nex2 melanoma cells. Starting on the 1^st^ day after tumor cell inoculation, active arazyme (3 mg/kg) was administered i.p. every other day for 21 days. Melanotic pulmonary nodules were counted using an inverted microscope on the 22^nd^ day. Control group (n = 7), arazyme treated group (n = 5*)*. The average numbers of nodules and standard deviations are shown. (**B**) Representative lung images of untreated and treated animals. One of three independent experiments is represented. Scale bar, 5 mm. * p<0.05.

C57Bl/6 mice were challenged intravenously with 5×10^5^ B16F10-Nex2 murine melanoma cells and treated intraperitoneally with 3 mg/kg of active arazyme for a period of 21 days on alternate days. The control group received PBS. There was a significant reduction in the number of metastatic pulmonary nodules after 22 days in arazyme-treated compared to untreated mice (Figure 1A and B). While five of seven animals in the control group showed around 200 pulmonary nodules, all animals in the treated group showed less than 20 nodules. This strong inhibition of tumor metastasis was observed in all 3 independent experiments performed.

### Active, but not heat-inactivated arazyme, displays a cytostatic effect on murine and human tumor cells *in vitro*

**Figure 2:**
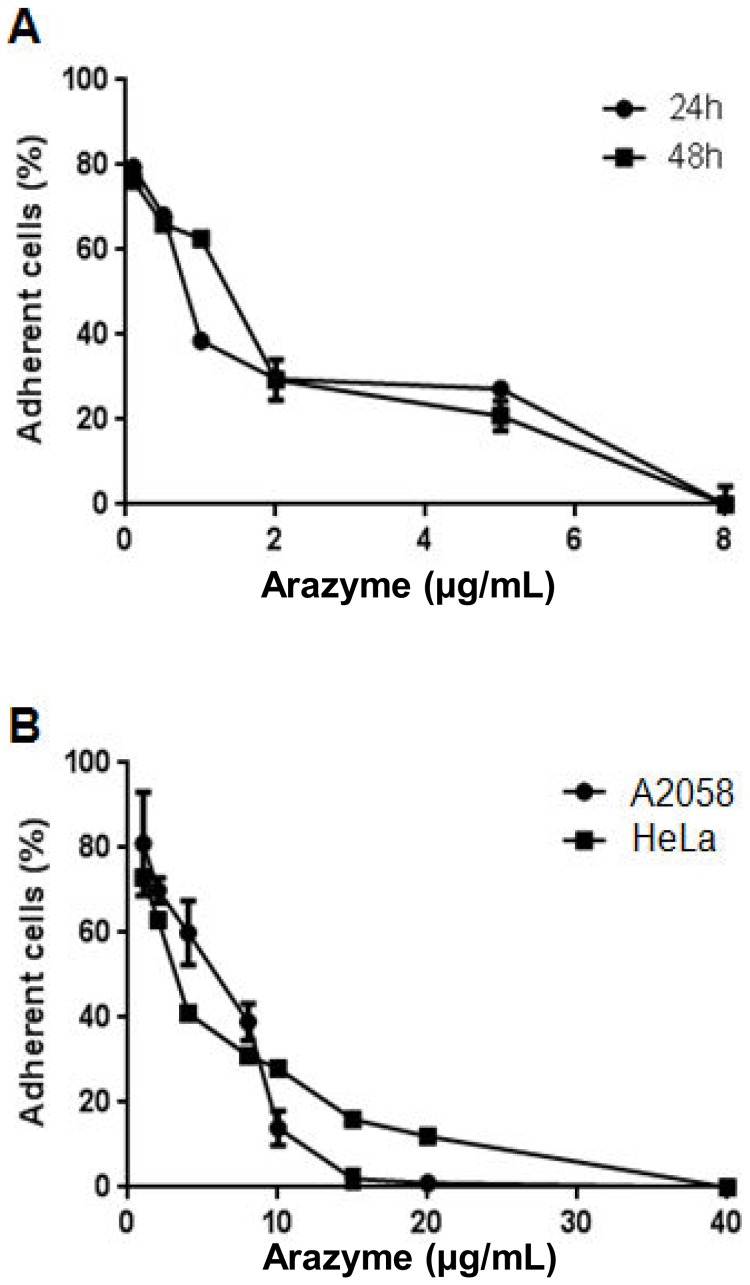
Active arazyme has a dose-dependent in vitro effect on B16F10-Nex2 murine melanoma and human tumor cells. (**A**) Murine melanoma B16F10-Nex2 cells were incubated with increasing concentrations of arazyme for 24 and 48 hours. (**B**) A2058 and HeLa tumor cells were treated with active arazyme for 48 hours. Cell viability was measured as described in materials and methods.

After *in vitro* incubation of murine melanoma B16F10-Nex2 cells with several doses of active arazyme, the supernatant was discarded and only viable adherent cells were counted with Trypan blue. It was observed that arazyme reduces the number of viable adherent cells in a dose-dependent way. At a concentration of 8 µg/mL, arazyme was able to detach all adherent cells after 24 hours (Figure 2A).

Arazyme was also able to reduce the number of viable adherent cells after 48 hours incubation in human melanoma A2058 and human uterine cervix carcinoma HeLa cells (Figure 2B).

**Figure 3:**
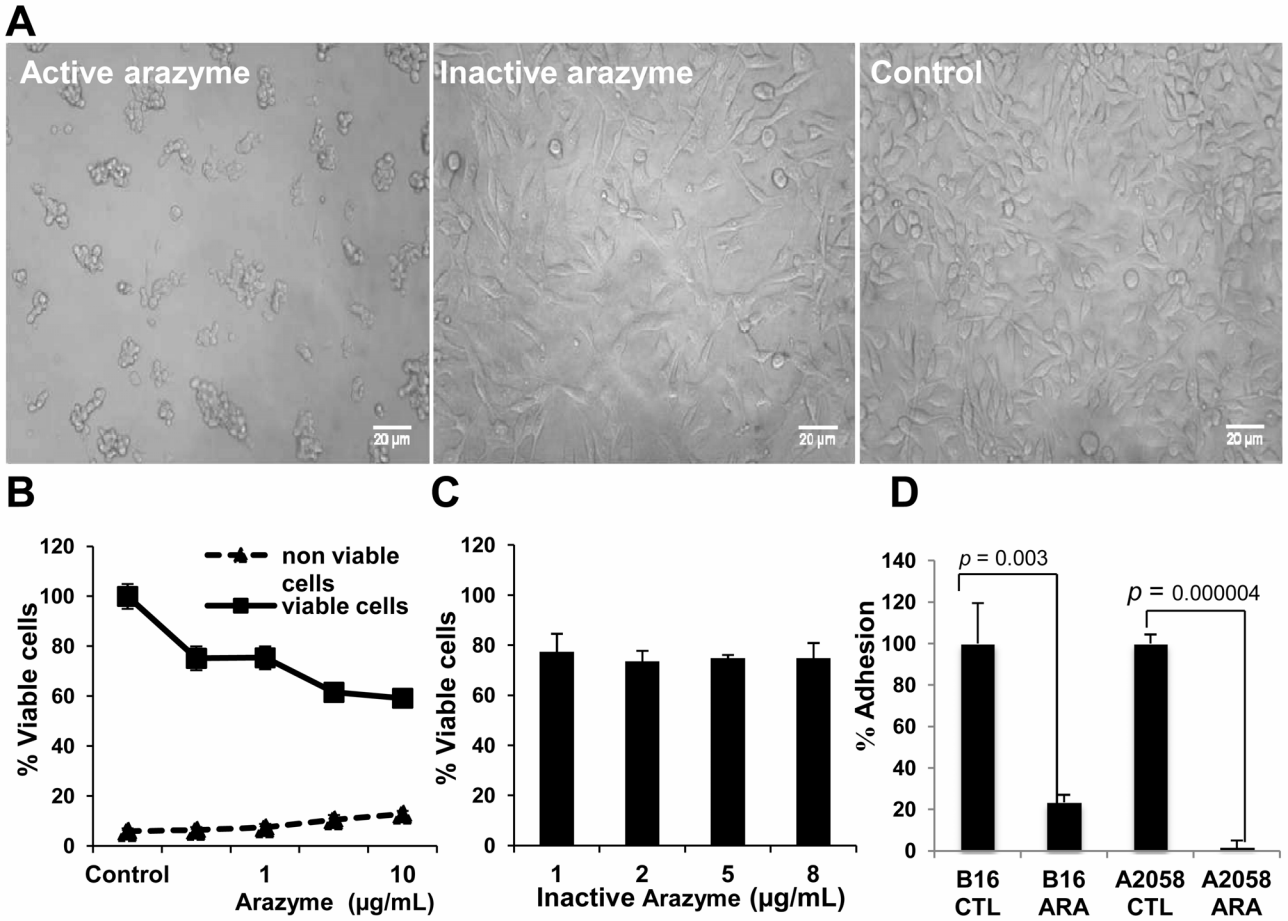
Active but not heat-inactivated arazyme has a cytostatic effect on murine melanoma B16F10-Nex2 cells. (**A**) Representative images of tumor cells treated for 24 hours with 8 µg/mL of active or heat-inactivated arazyme. Control, untreated cells. (**B**) B16F10-Nex2 cells were cultured for 72 hours, and different concentrations of active arazyme (0.5, 1, 5 or 10 µg/mL) were added on 0, 24 and 48 hours time points. The supernatant was carefully removed after plate centrifugation (10 min, 2,500 rpm) and fresh culture medium without arazyme was added. After 24 hours, cells were counted in presence of Trypan blue and the percentage of viable and non-viable cells was calculated compared to untreated control. (**C**) Tumor cells were treated with increasing concentrations of heat-inactivated arazyme for 48 hours. Viable cells were counted in presence of Trypan blue. (**D**) B16F10-Nex2 and A2058 cells (5×10^4^ cells/well) were treated with arazyme (10 µg/mL) for 1 hour, plated on 96-wells plate, and after incubation for 3 hours, non-adherent cells were removed by a PBS rinse and adherent cells were stained. Data are representative of three independent experiments. p values are shown in the figure.

B16F10-Nex2 cells treated with a high dose of active arazyme (8 µg/mL) for 24 hours showed intense morphology alterations, such as loss of substrate adhesiveness and cluster formation (Figure 3A). This effect was dependent on the metalloprotease activity of arazyme, since heat-inactivated protease neither induced alterations on cell morphology (Figure 3A) nor interfered with cell viability (Figure 3C).

Surprisingly, it was observed that most cells in the clusters present in the culture supernatant after arazyme treatment were viable, hence not stained by Trypan blue, and we next verified the ability of these viable cells to proliferate after protease removal from the culture medium. B16F10-Nex2 cells were treated with several doses (0.5 to 10 µg/mL) of active arazyme for 72 hours, and to avoid loss of enzyme activity by degradation in the culture medium, arazyme was added at 0, 24 and 48 hours incubation times. Plates were then centrifuged and the arazyme-containing supernatant was carefully removed, leaving adherent and non-adherent cells at the bottom of the well. Fresh culture medium without arazyme was added, and cultures were further incubated for additional 48 hours in the absence of the protease. After this final incubation, the number of viable cells was slightly reduced in arazyme-treated cultures compared to the untreated control, and the number of non-viable cells was less than 10% in treated and untreated cultures (Figure 3B).

To demonstrate that arazyme interferes with tumor cell adhesion, murine B16F10-Nex2 and human A2058 melanoma cells were treated *in vitro* with arazyme for 1 hour, plated and incubated for additional 3 hours. Non-adherent cells were then removed, and attached cells were colorimetrically quantified. Adhesions of B16F10-Nex2 and A2058 cells were significantly reduced by 80% and more than 90%, respectively (Figure 3D).

These results suggest that active arazyme is not directly cytotoxic in human and murine tumor cells, but instead, has a cytostatic effect, interfering with tumor cell adhesion to ECM and neighbouring cells.

### Arazyme cleaves tumor cell surface CD44 and reduces *in vitro* and *in vivo* cell invasion

**Figure 4:**
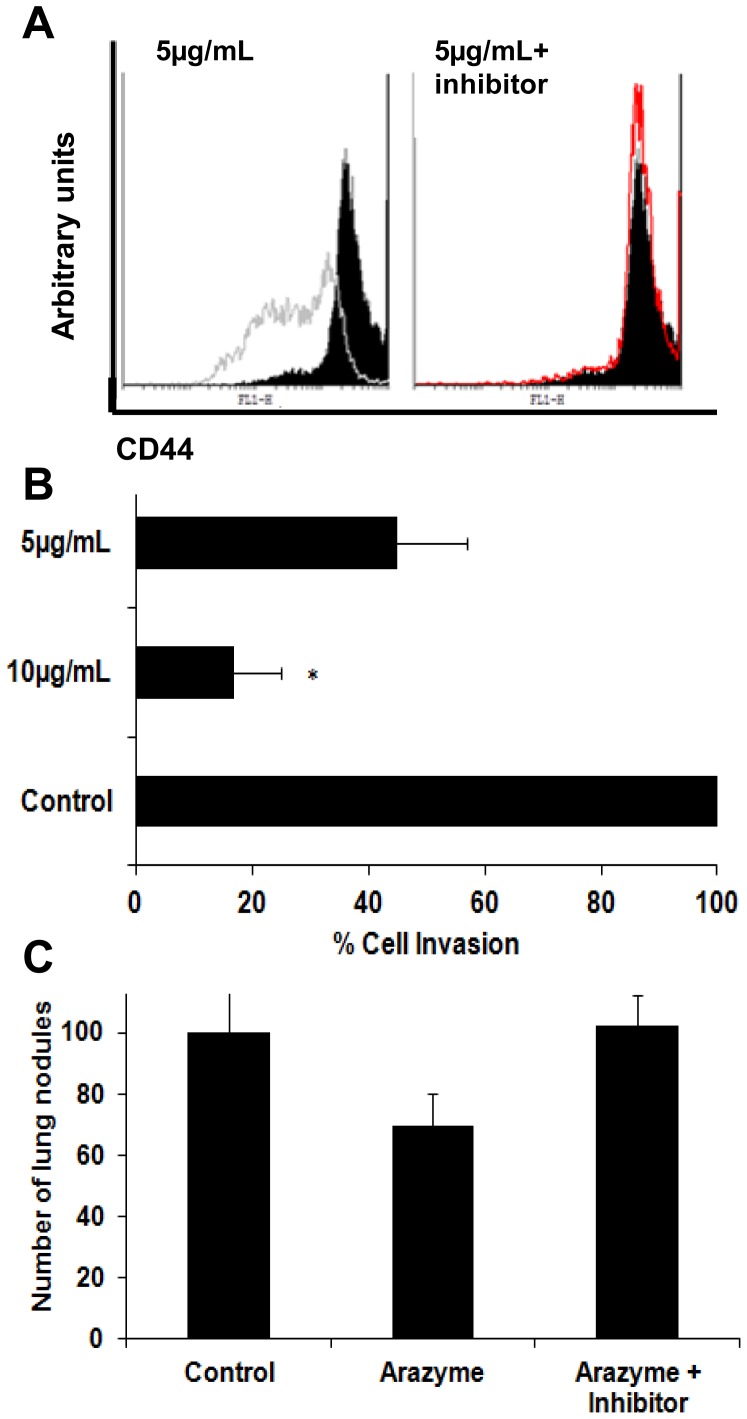
Metalloprotease activity of arazyme reduces CD44 expression on tumor cell surface, and reduces *in vitro* and *in vivo* the invasion of B16F10-Nex2 melanoma cells. (A) B16F10-Nex2 cells were treated for 1 hour with 5 µg/mL of arazyme with or without *ortho-*phenantrolyne (2 mM) and were then incubated with FITC-conjugated anti-murine CD44 antibody. The full peak (black) represents CD44 expression in untreated tumor cells and the open area shows CD44 expression after treatment. (B) B16F10-Nex2 cells were previously incubated with arazyme (5 or 10 µg/mL for 1 hour) and the ability of these cells to invade matrigel was measured as described in materials and methods. Results of three independent experiments were grouped. (C) B16F10-Nex2 melanoma cells were previously incubated for 1 hour at 37°C with 20 µg/mL of active arazyme, 2 mM *ortho*-phenantrolyne-inactivated arazyme or saline solution. C57Bl/6 mice (5 animals/group) were i.v. inoculated with 3×10^5^ treated tumor cells and lung nodules were counted after 15 days.*, p≤0.05.

Proteases have been shown to influence the expression of adhesion molecules on tumor cell surface, and one of such molecules, CD44, is highly expressed in tumor cells [22], [23]. CD44 has a role in B16F10-Nex2 cells migration and invasion [11], [24]. After 1 hour incubation with 5 µg/mL of active arazyme, a significant reduction on surface CD44 was detected on B16F10-Nex2 cells (Figure 4A). This effect was abolished when the catalytic activity of arazyme was inhibited by *ortho*-phenantroline (Figure 4A). Reduction of CD44 molecules on the cell surface was also observed in human melanoma A2058 cell line after arazyme treatment (Figure S1A). The reduction on the tumor cell surface CD44 was due to the catalytic activity of the protease, since CD44 gene expression was not affected by arazyme treatment in both tumor cell lines (Figure S1B).

Arazyme treatment reduced the invasiveness of B16F10-Nex2 melanoma cells *in vitro* as well as *in vivo*. For *in vitro* testing, Matrigel-coated transwell chambers and fetal calf serum as haptotactic stimulus were used. Matrigel contains the extracellular matrix of murine sarcoma cell line, and cell penetration using this material simulates the *in vivo* tumor cell invasion of connective tissue and basal membrane. A dose-dependent reduction in B16F10-Nex2 cell invasion after incubation with 5 or 10 µg/mL of arazyme was observed (Figure 4B).

Additionally, B16F10-Nex2 cells were incubated for 1 hour at 37°C with active arazyme in the presence or not of *ortho*-phenantroline, washed with PBS for enzyme/inhibitor removal and injected endovenously in C57Bl/6 animals. After 13 days, the number of lung nodules was counted. Treatment of melanoma cells with arazyme reduced significantly the number of lung nodules formed *in vivo*, and this effect was reversed by the metalloprotease-inhibitor (Figure 4C).

### Arazyme induces monospecific polyclonal antibodies that are cytotoxic to tumor cells *in vitro*, cross-react with tumor MMP-8, and are protective after passive transfer *in vivo*

Guimarães-Ferreira et al [11] demonstrated that murine antibodies induced after treatment with fastuosain or bromelain, plant-derived proteolytic products, cross-reacted in ELISA with cathepsins B and L, and were lytic to B16F10-Nex2 cells *in vitro*, reacting with surface and cytoplasmic components expressed by these cells.

**Figure 5:**
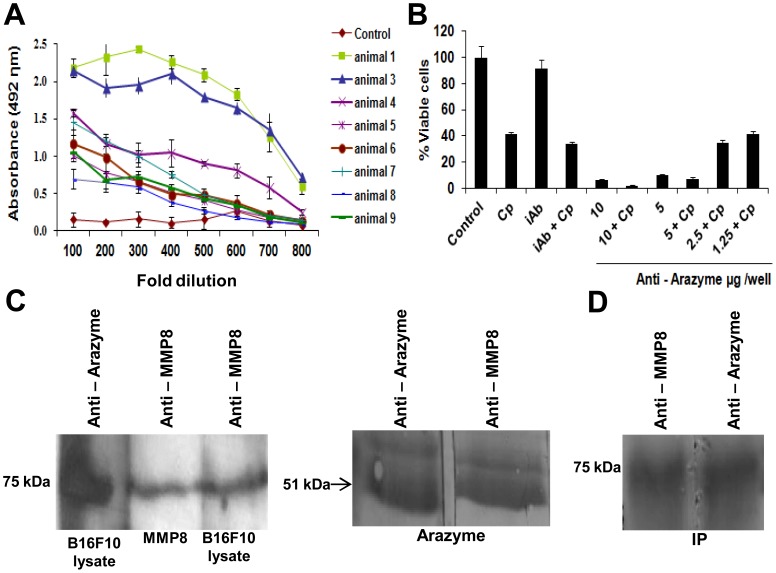
Arazyme treatment induces protease-specific antibodies that cross-react with matrix metallopeptidase 8 (MMP-8) and are cytotoxic to B16F10-Nex2 cells. (A) Serum (100^−1^ to 800^−1^ dilutions) from i.p arazyme-treated or untreated C57Bl/6 mice were analyzed by ELISA for arazyme-specific antibodies as described in materials and methods. Animals are represented individually. (B) *In vitro* cytotoxicity of anti-arazyme antibodies. Murine arazyme-specific protein G-purified IgG (1.25 to 10 µg/well) was incubated in the presence or absence of guinea-pig complement for 12 hours, viable cells were counted in presence of Trypan blue and compared to the untreated control. Cp, Complement and iAb, irrelevant antibody.*, p≤0.05. (C) B16F10-Nex2 cell extract, purified arazyme or recombinant MMP-8 were electrophoretically separated, blotted onto nitrocellulose membrane and revealed with rabbit anti-arazyme or monoclonal anti-MMP-8 antibodies. (D) B16F10-Nex2 cell extract was immunoprecipitated with Ab anti-arazyme, and the complex was collected with Sepharose-Protein-G beads. Western blotting of the precipitated sample was revealed with rabbit anti-arazyme or monoclonal anti-MMP-8 antibodies.

Anti-arazyme monospecific antibodies were raised in C57Bl/6 mice, analyzed in ELISA (Figure 5A) and the IgG fraction purified in a protein G column. It proved to be highly cytotoxic *in vitro* in B16F10-Nex2 melanoma cells, in a dose-dependent manner, even in the absence of complement (Figure 5B).

Polyclonal antibodies were also obtained at high titers in rabbits immunized s.c. with arazyme using alum as adjuvant (Figure S2A). Purified rabbit IgG were also directly cytotoxic in B16F10-Nex2 cells, and the effect increased with complement (Figure S2B).

To identify the possible targets in murine melanoma cells for the antibodies raised against arazyme, the protein structure of the enzyme was compared to other metallopeptidases. Based on their amino acid sequences four metallopeptidases families are recognized: astacins, serralysins, adamalysins and matrixins, collectively classified as members of the metzincin superfamily. Using BLAST program (http://blast.ncbi.nlm.nih.gov/Blast.cgi), it was observed that arazyme (accession number AAX21094.1) showed similarity ranging from 37 to 46% with murine matrix metalloproteases (MMP) 1, 7, 8, 11 and 20 (Table 1). Moreover, as a member of serralysin family, arazyme contains an elongated zinc-binding motif, HEXXHXXGXXH, and a methionine-turn [17], [25]. Topologically, MMP-8 (neutrophil collagenase) has a structure similar to serralysins [25], [26] including arazyme. We therefore explored whether the anti-arazyme antibodies would recognize tumor-cell associated MMPs.

**Table 1:** Similarity between murine (*Mus musculus*) matrix metalloproteases and arazyme.

Protein	Acession number	Maximum score	Query coverage
MMP-1	NP_114396.3	46.2	37%
MMP-7	NP_034940.2	42.0	32%
MMP-20	NP_038931.1	42.0	33%
MMP-8	NP_032637.3	38.9	33%
MMP-11	NP_032632.1	37.4	46%

Maximum scores over 30% are showed.

Reactivity of anti-arazyme antibodies and monoclonal antibodies to MMPs 1, 7, 8, 11 and 20 to total B16F10-Nex2 cell lysate was compared in Western-blot. Monoclonal antibodies against MMP-2 and MMP-9 were also included in this experiment because both have a role in melanoma development [27], [28].

Monoclonal antibodies to MMP-8 recognized a component of 75 kDa in the B16F10-Nex2 lysate, with the same molecular weight of recombinant MMP-8 (Figure 5C). Further, the same component of 75 kDa was recognized by anti-arazyme Abs in murine B16F10-Nex2 tumor lysate (Figure 5C) and in human melanoma A2058 and human breast carcinoma SKBR3 cell lysates (Figure S3). Monoclonal anti-MMP-8 antibodies cross-reacted with purified arazyme (Figure 5C).

Monoclonal antibodies against the other MMPs tested showed no specific reactivity with components of B16F10-Nex2 lysate, but recognized the respective recombinant proteases used as control (data not shown).

The cross reactivity of anti-arazyme with murine MMP-8 was confirmed by immunoprecipitation. B16F10-Nex2 lysate was incubated with anti-arazyme Abs, the complex was precipitated with Sepharose-Protein G beads, and the eluted 75 kDa component reacted with rabbit anti-arazyme Abs as well as with anti-MMP-8 antibodies (Figure 5D).

**Figure 6:**
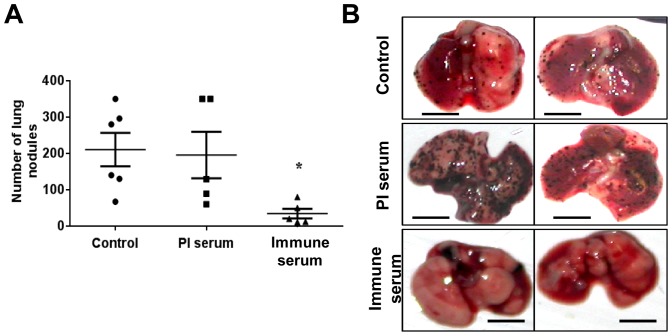
Passive transfer of rabbit anti-arazyme polyclonal antibodies inhibited murine melanoma metastasis. (**A**) C57Bl/6 mice were i.v. injected with 3×10^5^ B16F10-Nex2 melanoma cells. Starting on the 1^st^ day after tumor cell inoculation, 5 doses of 300 µL of rabbit pre-immune (**PI serum**) or the same volume of rabbit arazyme-specific immune serum was administered i.p. every other day. Melanotic pulmonary nodules were counted using an inverted microscope thirteen days after tumor cell inoculation. Untreated control group, n = 7 and pre-immune/immune serum treated groups, n = 5.*, p≤0.05. (**B**) Representative images of lungs from animals treated as described in (A). Scale bar, 5 mm.

Finally, we evaluated the ability of these anti-arazyme antibodies to inhibit melanoma metastasis *in vivo*. C57Bl/6 mice were challenged i.v. with 3×10^5^ B16F10-Nex2 murine melanoma cells and treated i.p. with 5 doses of 300 µL of PBS (control group), rabbit pre-immune or rabbit anti-arazyme serum, in alternate days. Thirteen days after tumor inoculation, the number of lung metastatic nodules was counted (Figure 6). Mice treated with anti-arazyme serum were significantly protected against melanoma metastasis development with an average number of lung nodules at least 4 times lower than that in untreated animals.

## Discussion

Despite all the efforts for metastatic cancer control during the last decades, this is still a very aggressive and deadly disease. Novel synthetic chemotherapeutic agents currently used in the clinics do not show the expected effectiveness, and there is increasing evidence for the potential for nature-derived compounds on cancer prevention and therapy. Many anticancer agents available today are plant-derived natural products or their analogs, but still new treatment alternatives are necessary to overcome the pitfalls in cancer treatment [29], [30].

Recently, bacterial-derived products have being considered promising therapeutic strategies to modulate the immune response in various human diseases, and several compounds with immunostimulatory properties were already described. Examples of these compounds are bacterial-produced Toll-like receptor (TLR) ligands and uracil, able to activate the innate immune response and to modulate intestinal immunity, respectively [31], [32].

Tumor-secreted proteases are in general associated with tumor progression, an effect attributed to ECM degradation, thus facilitating the process of tumor cell invasion and metastasis [33], [34]. On the other hand, some studies have shown that proteases may exert tumor suppressor effects. Exogenously added plant-derived fastuosain, bromelain or papain, and mammalian trypsin or chymotrypsin, show antitumor properties in preclinical cancer models [13]. Even some endogenous proteinases have showed antitumor effects, as MMP-8, MMP-12, MMP-19, MMP-26, caspase 3, caspase 5 and 6, prostasin, ADAMTS1, among others [33]–[35]. The molecular mechanisms by which these proteases play antitumor activities are not completely unraveled.

In the present work, we show that arazyme, a bacterial metalloprotease with commercial value displays antimetastatic property in the preclinical model of B16F10 melanoma. Intraperitoneal treatment with this metalloprotease significantly reduced the number of pulmonary nodules following intravenous injection of melanoma cells. Interestingly, arazyme did not inhibit subcutaneous B16F10-Nex2 melanoma development, when used in a therapeutic protocol administered i.p. (data not shown).

*In vitro*, arazyme showed a dose-dependent cytostatic effect in B16F10-Nex2 cells, reducing cell adhesion by reducing CD44 molecules on tumor cell surface. This caused detachment of adherent tumor cells, released from the matrix substrate to form clusters of live cells in the culture supernatant. When the active protease was removed from the culture, tumor cells adhered again and proliferated. Reduction of CD44 molecules on cell surface dependend on the proteolytic activity of arazyme, since the heat-inactivated protease was unable to cause the same effect. Moreover, CD44 mRNA expression was not altered by arazyme treatment of cells. Reduction of CD44 on tumor cells rendered inhibition of Matrigel invasion by these cells, and arazyme-treated tumor cells showed reduced lung colonization after endovenous inoculation. Inhibition of arazyme proteolytic activity by heating or by *ortho*-phenantroline abolished these effects. The cytostatic effect of arazyme, reduction of CD44 molecules although with normal CD44 mRNA expression, and reduction on cell adhesion *in vitro* was also seen on human tumor cells.

Proteolytic enzymes may act on adhesion molecules that play an important role in tumor development and metastasis [36]. Because of the intense crosstalk between the molecules that compose the ECM and the effects on tumor regulation, different therapeutic approaches have been used targeting members of the integrin family, CD44 and MMPs [37]–[39].

CD44 is a glycoprotein receptor that binds to extracellular hyaluronic acid (HA), important in cell-cell and cell-ECM adhesion [40], [41]. Different tumor cells have high expression of CD44, which plays a critical role in tumor progression, because degradation of HA facilitate tumor cell invasion and the spread of cancer [42], [43]. The lower expression of CD44, by using blocking antibodies, proteases or specific ligands, may regulate tumor metastasis [40], [44], [45]. Seemingly, the reversible cytostatic effect of arazyme on B16F10-Nex2 cells involves proteolysis of CD44 that reduces the adhesion of tumor cells to the matrix substrate. Detached cells form cell clusters in the culture supernatant. These tumor cells, however, are resistant to anoikis and can survive for some time [46]–[48]. After enzyme removal, CD44 *de novo* synthesis reconstitutes its surface expression, and cell cycle resumes after tumor cell adhesion.

We have previously shown that fastuosain, a cysteine-protease from *Bromelia fastuosa* protected mice against murine melanoma B16F10-Nex2 subcutaneous development, mainly by reduction of CD44 expression, which led to decreased tumor cell invasion [11]. It was also demonstrated that inoculation of bromelain and fastuosain in mice induced the production of polyclonal enzyme-specific immunoglobulins that cross-reacted with cathepsins B and L of murine origin that are highly expressed in B16F10 cells [11]. These bromelain- and fastuosain-specific antibodies were cytotoxic *in vitro* to B16F10-Nex2 cells, suggesting that *in vivo*, the protective effect of these cysteine proteases against murine melanoma could be partially due to the activity of these induced antibodies.

Presently, we show that *in vitro* monospecific polyclonal anti-arazyme antibodies are cytotoxic to tumor cells in a complement-independent manner. Most importantly, passive transfer of purified IgG fraction was protective *in vivo*, reducing significantly the number of lung metastatic nodules in B16F10-Nex2 challenged mice. Interestingly, the arazyme-specific immunoglobulins cross-react with MMP-8 expressed in B16F10-Nex2 cells, and may interfere with tumor cell development *in vivo*. Anti-arazyme antibodies recognized components of same molecular weight in human tumor cell lysates, suggesting that these antibodies can be cytotoxic also to human tumors.

MMP-8, also known as collagenase-2 or neutrophil collagenase, is a protease that is mainly produced by neutrophils, but is also involved in several pathologies, including cancer [49], [50]. The role of this MMP is not completely established in tumor development. While some studies show the relationship between MMP-8 and tumor progression, there are others associating MMP-8 with tumor suppression, especially in melanoma [51]–[53]. Experiments using MMP-8 genetically deficient mice showed the increased incidence of skin cancer, and overexpression of MMP-8 in melanoma cells decreased tumor invasion and up regulated the adhesion of tumor cells to ECM [50], [54]. The protective role of MMP-8 against melanoma development was reinforced in a study showing that MMP-8 is frequently mutated in melanoma [55]. Therefore, the wild type-proteolytic active MMP-8 displays tumor suppressive effects due to inhibition of tumor migration, invasion and metastasis, whereas mutant MMP-8 is inactive, allowing melanoma progression [45]–[56].

Recently, Kim and collaborators [57] showed that arazyme inhibited the secretion of inflammatory mediators by human immortalized keratinocytes (HaCaT) and monocytic (THP-1) or eosinophilic (EoL-1) tumor cell lines *in vitro*. These cells were stimulated, and treatment with arazyme reduced the expression of IL-6, IL-8, MCP-1/CCL-2 and TARC/CCL-17, suggesting that arazyme may also have an immunomodulatory effect *in vivo*, that should be investigated.

In summary, we describe here the strong antitumor effect of a bacterial-derived natural product, the metalloprotease arazyme, using the metastatic preclinical model of murine melanoma B16F10-Nex2. The active protease reduced CD44 molecules at tumor cell surface interfering with cell adhesion, causing a cytostatic effect *in vitro*. Arazyme removal from culture allowed tumor cells to adhere again and proliferate, since they were resistant to anoikis. In addition, arazyme induced the production of protease-specific IgGs that cross-reacted with tumor MMP-8. These antibodies were cytotoxic *in vitro* to melanoma cells, and *in vivo* reduced melanoma lung metastasis. We conclude that the strong antitumor activity of arazyme against murine B16F10-Nex2 metastatic melanoma could be due to the direct activity of the active protease on tumor cells in combination with the cytotoxic arazyme-specific immunoglobulins cross-reactive with MMP-8 expressed by tumor cells, making this bacterial metalloprotease a promising candidate for metastatic disease control.

## Supporting Information

Figure S1 **Metalloprotease activity of arazyme reduces CD44 molecules on human melanoma cell surface, but not interferes with CD44 gene expression.** (**A**) A2058 cells were treated for 1 hour with 20 µg/mL of arazyme in presence or not of the inhibitor *ortho*-phenantroline, washed and incubated with FITC-conjugated anti-human CD44 antibody. Open peaks represent CD44 expression on untreated tumor cells and solid curves show CD44 expression after arazyme treatment. (**B**) RT-PCR showing CD44 relative gene expression on A2058 and B16F10 cells treated for 1 hour with arazyme (10 µg/mL). GAPDH and HPRT were used as the constitutive expression control respectively for A2058 and B16F10-Nex2 cells.
(TIF)

Figure S2 **Arazyme treatment induces rabbit protease-specific antibodies that reduce B16F10-Nex2 cells viability.** (**A**) Serum (200^−1^ to 256000^−1^ dilutions) from arazyme-immunized rabbits was analyzed by ELISA as described in materials and methods. (**B**) *In vitro* cytotoxicity of murine anti-arazyme antibodies: Rabbit policlonal arazyme-specific protein G-purified IgG (2.5–20 µg/well) was incubated in the presence or absence of guinea-pig complement for 12 hours, viable cells were counted in presence of Trypan blue and percentage was calculated compared to untreated control. **Cp**, Complement.*, p≤0.05, compared to Control.
(TIF)

Figure S3 **Arazyme-specific antibodies recognize a 75 kDa component in B16F10-Nex2 and human tumor cells lysate.** B16F10-Nex2, SKBR3 and A2058 cell extract (40 µg), were electrophoretically separated, blotted onto nitrocellulose membrane and revealed with rabbit anti-arazyme antibodies (1∶200).
(TIF)
