# Supplementary figures and images for: A Natural Bacterial-Derived Product, the Metalloprotease Arazyme, Inhibits Metastatic Murine Melanoma by Inducing MMP-8 Cross-Reactive Antibodies
Source: PLoS One. 2014 Apr 30;9(4):e96141. doi: 10.1371/journal.pone.0096141 (PMC4005744; doi:10.1371/journal.pone.0096141)

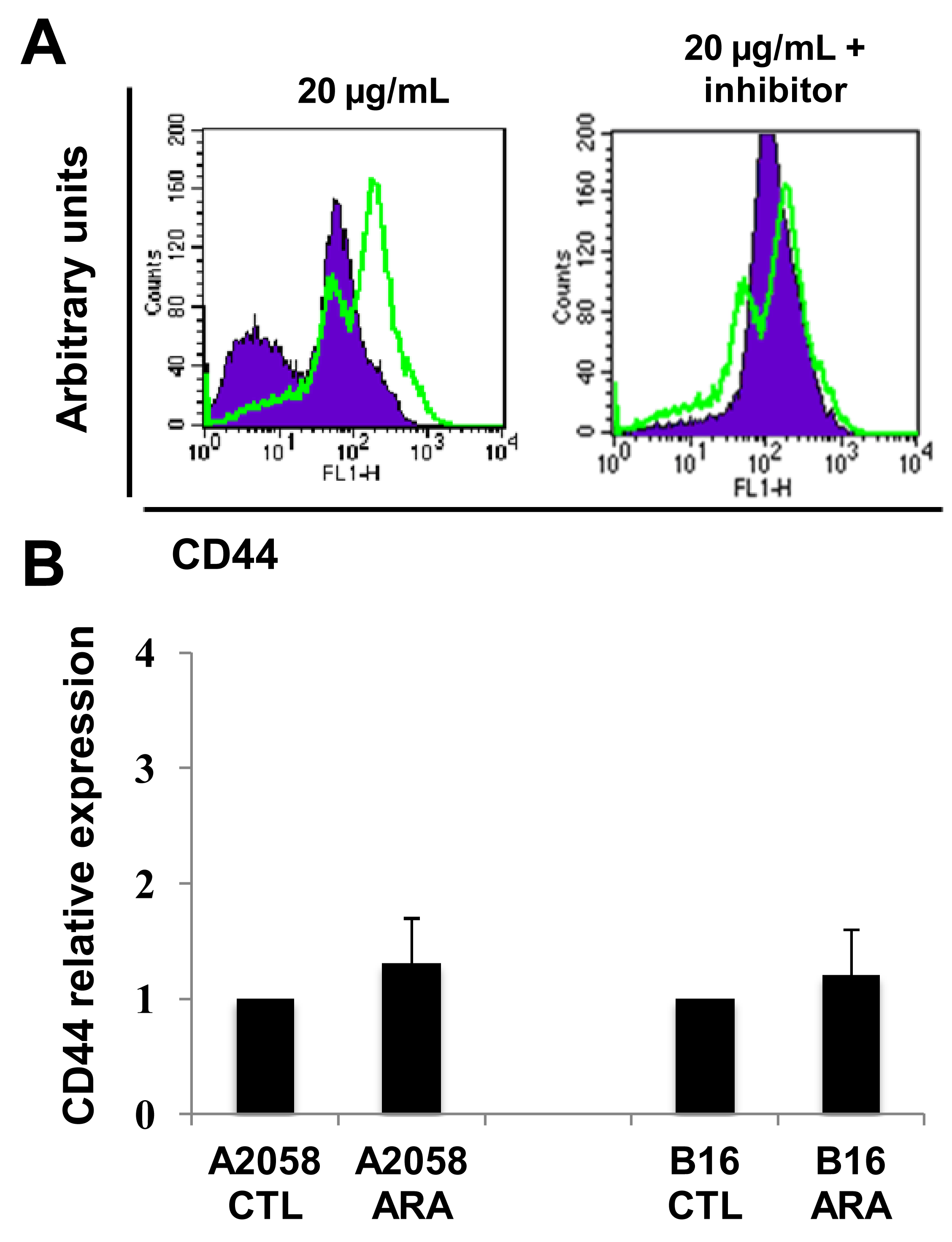

Supplement: Figure S1 — Metalloprotease activity of arazyme reduces CD44 molecules on human melanoma cell surface, but not interferes with CD44 gene expression. (A) A2058 cells were treated for 1 hour with 20 µg/mL of arazyme in presence or not of the inhibitor ortho-phenantroline, washed and incubated with FITC-conjugated anti-human CD44 antibody. Open peaks represent CD44 expression on untreated tumor cells and solid curves show CD44 expression after arazyme treatment. (B) RT-PCR showing CD44 relative gene expression on A2058 and B16F10 cells treated for 1 hour with arazyme (10 µg/mL). GAPDH and HPRT were used as the constitutive expression control respectively for A2058 and B16F10-Nex2 cells. (TIF) [file pone.0096141.s001.tif]

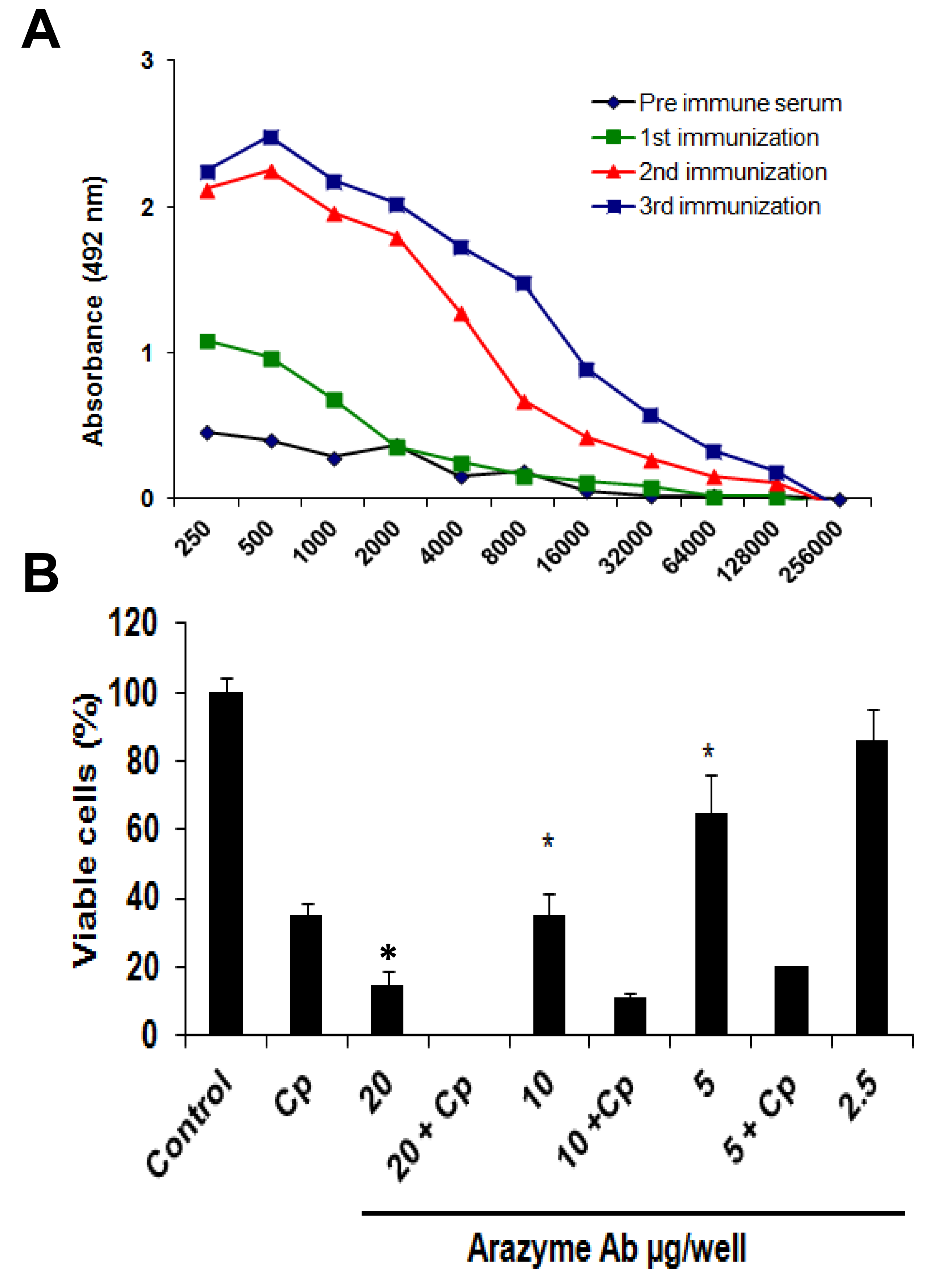

Supplement: Figure S2 — Arazyme treatment induces rabbit protease-specific antibodies that reduce B16F10-Nex2 cells viability. (A) Serum (200−1 to 256000−1 dilutions) from arazyme-immunized rabbits was analyzed by ELISA as described in materials and methods. (B) In vitro cytotoxicity of murine anti-arazyme antibodies: Rabbit policlonal arazyme-specific protein G-purified IgG (2.5–20 µg/well) was incubated in the presence or absence of guinea-pig complement for 12 hours, viable cells were counted in presence of Trypan blue and percentage was calculated compared to untreated control. Cp, Complement.*, p≤0.05, compared to Control. (TIF) [file pone.0096141.s002.tif]

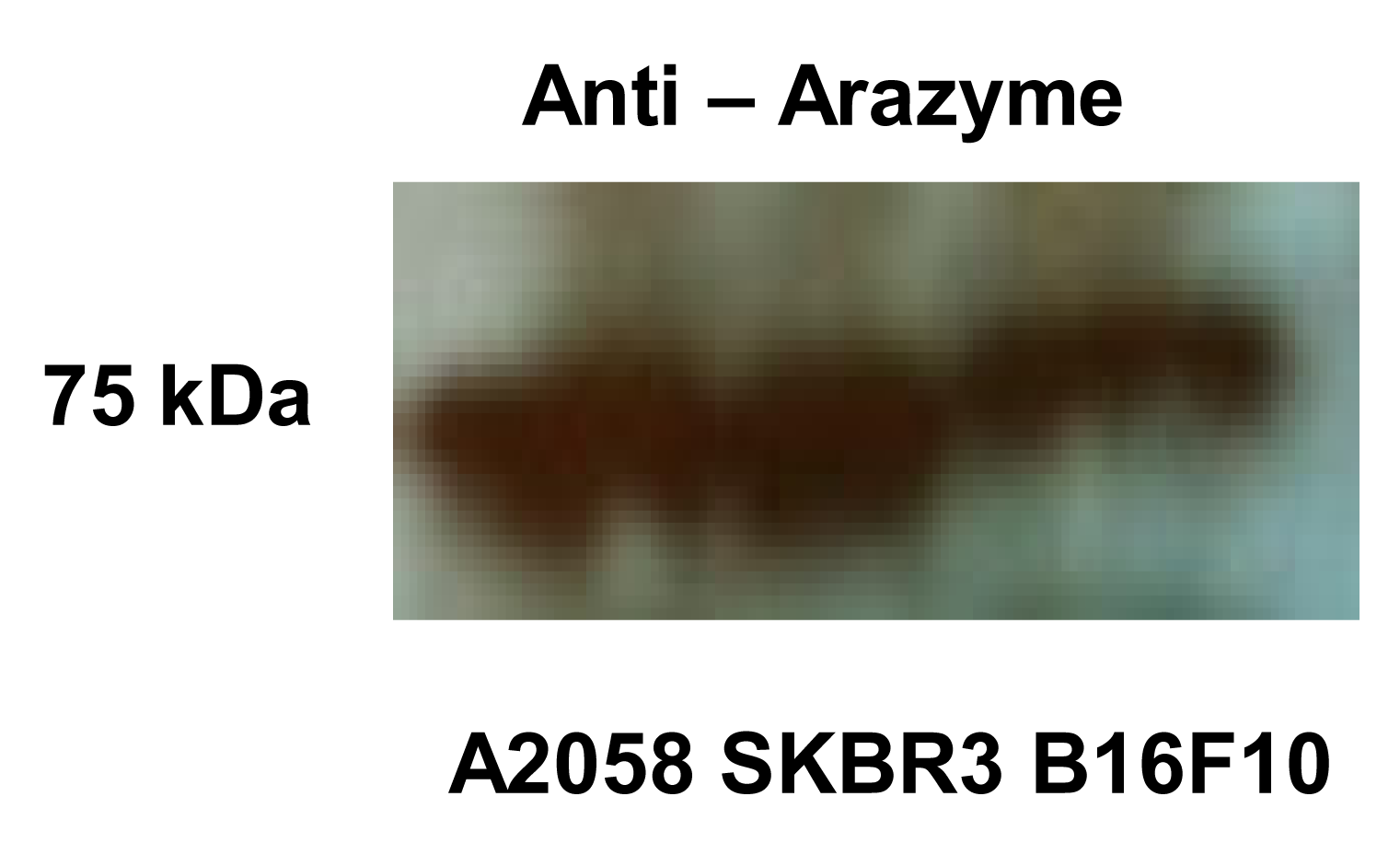

Supplement: Figure S3 — Arazyme-specific antibodies recognize a 75 kDa component in B16F10-Nex2 and human tumor cells lysate. B16F10-Nex2, SKBR3 and A2058 cell extract (40 µg), were electrophoretically separated, blotted onto nitrocellulose membrane and revealed with rabbit anti-arazyme antibodies (1∶200). (TIF) [file pone.0096141.s003.tif]
